# Supplementary material for: Conformal elasticity of mechanism-based metamaterials
Source: Nat Commun. 2022 Jan 11;13:211. doi: 10.1038/s41467-021-27825-0 (PMC8752823; doi:10.1038/s41467-021-27825-0)
Supplement: Supplementary file 1 — Supplementary Information [file 41467_2021_27825_MOESM1_ESM.pdf]

Supplementary Information

Supplementary Note 1. QUANTIFYING LOCAL DILATIONS AND SHEARS

To our knowledge, a parameter which quantifies what fraction of a *nonlinear* deformation is composed of local dilation, as opposed to local shear, has not been introduced previously. We therefore present a more detailed derivation in the context of Finite Strain Theory. This is an essential ingredient for supporting our hypothesis in the main text that nonuniform deformations of a dilational metamaterial are nonetheless composed primarily of local dilations.

The method described here applies to data describing deformation from a reference space $\mathbf{X}$ to a target space $\mathbf{x}(\mathbf{X})$, which are smooth enough to take derivatives. In the case of the RS metamaterial, the square centers provide a discrete approximation to a smooth deformation, which may then be used to define local material derivatives. More generally, one may use the lattice vectors of a material with repeating metastructure as material vectors to take these material derivatives. From these derivatives we assemble the deformation tensor

$$F_{ij}(\mathbf{x}) = \frac{\partial x_i}{\partial X_j}|_{\mathbf{x}} \,. \tag{1}$$

For a smooth deformation, this tensor contains all required information to quantify dilation and shear at each point in space.

Using the polar decomposition theorem, we may write $\mathbf{F} = \mathbf{R}\cdot\mathbf{U}$, where $\mathbf{R}$ is a rotation and $\mathbf{U}$ is the symmetric "right stretch tensor". From this form, it is clear that an infinitesimal material vector in the reference space $\mathrm{d}\mathbf{X}$ will deform first via multiplication with $\mathbf{U}$, then will be rotated via $\mathbf{R}$ to reach a final state $\mathrm{d}\mathbf{x} = \mathbf{R}\cdot\mathbf{U}\cdot\mathrm{d}\mathbf{X}$. Since $\mathbf{U}$ is symmetric, there exists a local choice of coordinate system where $\mathbf{U}$ is diagonal and acts on the local elements $\mathrm{d}\mathbf{X}$ as a combination of just dilation and pure shear. The eigenvalues (λ_1, λ_2) of $\mathbf{U}$ are called the *principal stretches* and the local material dilation and shear magnitudes may be written intuitively in terms of them. An infinitesimal square sample of side lengths (ϵ, ϵ) oriented in the diagonal frame of $\mathbf{U}$ will transform into a rectangle with side lengths $(\lambda_1\epsilon, \lambda_2\epsilon)$ after deformation. Using this, the local dilation may be defined intuitively in terms of the area

$$d \equiv \frac{\Delta A}{A_0} = \mathrm{Det}[\mathbf{U}] - 1 \,, \tag{2}$$

where ΔA is the change in area of our infinitesimal element and $A_0 = \epsilon^2$ is the initial area in the reference space. For a similarly intuitive notion of shear, we turn to the anisotropy of the tensor $\mathbf{U}$. We define the nonlinear shear magnitude in terms of the difference of the principal stretches as $s^2 = (\lambda_1 - \lambda_2)^2$. We will see that this definition matches with conventional intuition for standard simple shear nonlinearly as well as general mixed linear shear. Importantly, this shear may be rewritten in terms of invariants of $\mathbf{U}$ as $s^2 = \mathrm{Tr}[\mathbf{U}]^2 - 4\mathrm{Det}[\mathbf{U}]$, and is therefore also a rotationally invariant quantity.

Since the stretch tensor is generally less convenient to extract numerically, we show that this local dilation and shear may also be written in terms of the trace and determinant of the metric (equivalently the "right Cauchy-Green deformation tensor"). The metric is defined as

$$C_{ij} = \frac{\partial x_k}{\partial X_i}\frac{\partial x_k}{\partial X_j} = (\mathbf{F}^{\mathrm{T}}\mathbf{F})_{ij} = (\mathbf{U}^{\mathrm{T}}\mathbf{U})_{ij} \,. \tag{3}$$

The coordinate system which diagonalizes $\mathbf{U}$ also diagonalizes $\mathbf{C}$, and it follows that the eigenvalues of the metric are the squared eigenvalues of $\mathbf{U}$. Using this locally diagonalized frame, we may extract general relations between rotationally invariant quantities

$$\begin{aligned} \mathrm{Det}[\mathbf{U}]^2 &= \mathrm{Det}[\mathbf{C}], \\ \mathrm{Tr}[\mathbf{U}]^2 &= \mathrm{Tr}[\mathbf{C}] + 2\sqrt{\mathrm{Det}[\mathbf{C}]} \,. \end{aligned} \tag{4}$$

Using these relations, we may write the more useful formulas

$$\begin{aligned} d &= \sqrt{\mathrm{Det}[\mathbf{C}]} - 1, \\ s^2 &= \mathrm{Tr}[\mathbf{C}] - 2\sqrt{\mathrm{Det}[\mathbf{C}]} \,, \end{aligned} \tag{5}$$

completing our recipe to extract the local dilation and shear magnitudes from the metric tensor.

That these relations in equation (5) discriminate conformal from non-conformal deformation is not entirely obvious. We therefore present them in alternative form by connecting to the complex formulation where the vector mapping $\mathbf{x} \to \mathbf{x}(\mathbf{X})$ is captured by the complex function $z \to f(z, \bar{z})$. In this case, the components of the metric may be conveniently rewritten as

$$\begin{aligned} C_{xx} &= |\partial_z f|^2 + |\partial_{\bar{z}} f|^2 + 2\mathrm{Re}\left[\overline{\partial_z f}\partial_{\bar{z}} f\right] \\ C_{yy} &= |\partial_z f|^2 + |\partial_{\bar{z}} f|^2 - 2\mathrm{Re}\left[\overline{\partial_z f}\partial_{\bar{z}} f\right] \\ C_{xy} = C_{yx} &= 2\mathrm{Im}\left[\overline{\partial_z f}\partial_{\bar{z}} f\right] \,, \end{aligned} \tag{6}$$

where $\partial_z = 1/2(\partial_x - i\partial_y)$ and $\partial_{\bar{z}} = 1/2(\partial_x + i\partial_y)$ are standard complex derivatives [1]. With these relations, our metrics of conformal and non-conformal become

$$\begin{aligned} d^2 &= \left[|\partial_z f|^2 - |\partial_{\bar{z}} f|^2 - 1\right]^2, \\ s^2 &= 4|\partial_{\bar{z}} f|^2 \,, \end{aligned}$$

where, remembering that the conformal condition is $\partial_{\bar{z}} f = 0$, the shear part now more transparently quantifies the non-conformal part of the deformation. While both dilation and shear are strain quantities, there is

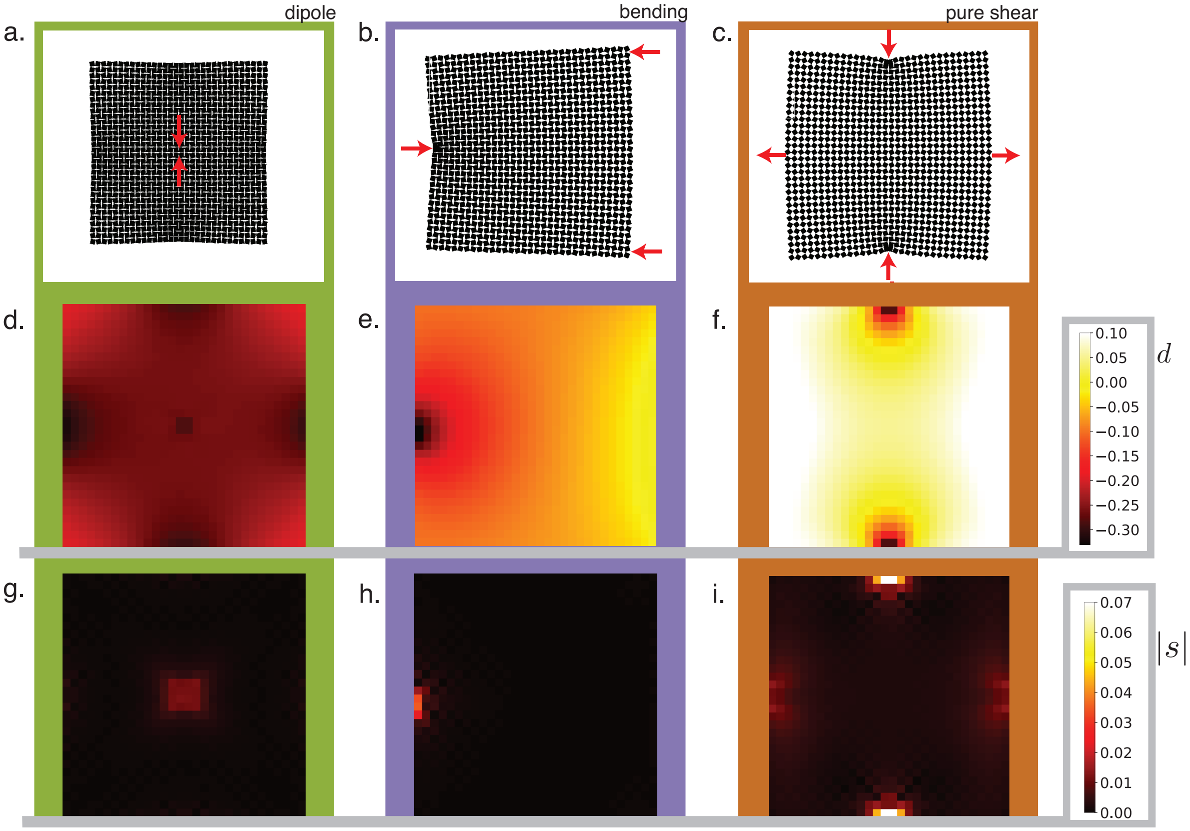

Supplementary Figure 1. Elastic RS-based structures deform with local dilation over shear. For each of the applied displacement loads (in columns according to **a**, **b**, **c**) from the main text, we display the spatial distributions of coarse dilation (d, **d**, **e**, **f**) and coarse shear magnitude ($|s|$, **g**, **h**, **i**) displaying a strong tendency of the RS-based metamaterial toward deformations that are locally dilation dominated.

some ambiguity in comparing them nonlinearly. The expressions used here, composed entirely in terms of invariants of $\mathbf{C}$, are constructed to be intuitive and geometric. In addition, rewriting

$$s^2 = (\partial_x u_x - \partial_y u_y)^2 + (\partial_x u_y + \partial_y u_x)^2 \qquad (7)$$

in terms of the components of the displacement $\mathbf{u} = \mathbf{x} - \mathbf{X}$ reveals that our definition of the shear matches with the combined magnitude of pure and simple shears (first and second terms in equation 7 respectively) from linear strain theory. Note that, in the linear limit, these strain measures satisfy $1/2(d^2 + s^2) = \epsilon_{ij}\epsilon_{ij}$. While alternate definitions of shear may be identified which also satisfy this in the linear limit, we have explored these and they do not qualitatively change the results presented here. Finally, for a conventional simple shear $\mathbf{u} = \gamma y \hat{x}$, our formula reassuringly recovers $s^2 = \gamma^2$ to nonlinear order.

As shown in Supplementary Fig. 1, these formulae reveal that the typical magnitude of local dilation is much larger than that of local shear. While the shear may become large in small regions near the applied loads and where the dilation changes over short distances, dilation still dominates by nearly an order of magnitude at the worst. Nonuniform, nonlinear deformations of the RS-based mechanism in force-balance may then indeed be composed of local dilations with comparatively very little shear.

Supplementary Note 2. LEAST-SQUARES METHOD TO EXTRACT THE NEAREST CONFORMAL MAP FROM DATA

We now identify a simple fitting method of locating the closest conformal map describing a discrete set of displacement data points. Per Supplementary Note 1, the discrete data should be chosen carefully to approximate a smooth deformation. For lattice materials, this means tracking identical points in neighboring unit cells. For the RS-based metamaterial, we can choose the centers of the square elements.

The method consists of first noting that a general conformal map $f(z)$ on a simple finite planar domain with no holes (i.e. simply-connected) may be expressed precisely

as an analytic function with a series expansion

$$f(z) = \sum_{n=0}^{\infty} C_n z^n \, . \tag{8}$$

In contrast, a generic non-conformal deformation must be expressed as a double sum over all products of z and the complex conjugate $\bar{z}$. The complex expansion coefficients C_n provide a complete description of the map and solving for them is therefore the goal of our analysis. Finding the nearest conformal map is a matter of minimizing the square error function

$$Err \equiv \sum_{i=1}^{n_p} |f_i - f(z_i)|^2 \, , \tag{9}$$

where the sum runs over the n_p discrete data points $z_i \to f_i$. Inserting the form of $f(z)$ (equation (8)) and minimizing with respect to the C_n, we find

$$\sum_{n=1}^{n_c} A_{mn} C_n = \sum_{i=1}^{n_p} f_i \bar{z}_i^m \, , \tag{10}$$

where we have defined the matrix

$$A_{mn} = \sum_{i=1}^{n_p} \bar{z}_i^m z_i^n \tag{11}$$

and the coefficients are now cut off at a maximum n_c. This effectively reduces the least-squares error analysis to a straightforward linear algebra problem, which is readily solved. We note that the cutoff n_c should be chosen to be much less than the n_p data points, to avoid over-fitting.

To evaluate the accuracy of the fit, we define a function

$$\Delta^2[u(z)] \equiv \frac{\sum_{i=1}^{n_p} |u_i - u(z_i)|^2}{\sum_{i=1}^{n_p} |u_i|^2} \, , \tag{12}$$

where $u_i = f_i - z_i$ is the observed displacement data, $u(z) \equiv f(z) - z$ are the displacements proposed to fit the data. The functional $\Delta^2[\,]$, known as the fraction of variance unexplained, is then a general method to quantify the amount of the deformation captured by a candidate conformal function, which we use in the main text and in other sections of this SI. The method evaluates error using the displacements rather than positions in order to avoid a false inflation of the results at small deformation. Inserting the numerically solved closest fit into equation (12), we show in Supplementary Fig. 2 that only around 20 coefficients are needed to fit the deformations of the RS metamaterial before the improvement starts to become negligible. This is good, as the more coefficients that are included, the more computationally expensive the fitting problem becomes.

We remark that, in practice, application of this method works best if the coordinate system is first centered and rescaled so that the data points reside closer to unity away from the origin. This helps to avoid large and small number issues in the numerics, which includes higher and higher exponents of the positions as more coefficients are included.

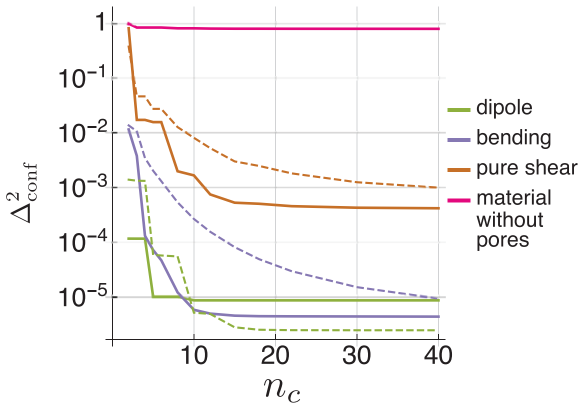

Supplementary Figure 2. The accuracy of the conformal map with the increasing number of coefficients allowed in the expansion equation (8). Solid lines correspond to fits with FEM data at the smallest strains, while the dotted lines are for the largest strains explored in the main text analysis. In comparison to the conventional elastic material in the absence of pores, the fitting quickly reaches very small errors $\Delta^2_{\rm conf}$ which is the function $\Delta^2[\,]$ evaluated for the particular candidate fit $u(z)$. For loads with discrete rotational symmetry, the errors go down in steps, since only every few coefficients may be non-zero.

This constitutes a fast and accurate method to extract the nearest conformal map from position data. Note that, while the method is reduced to linear algebra, no assumptions of linearity of the strains in either the deformation, or the fitting map, have been made. Indeed, this method shows the nonlinear deformations of the RS metamaterial are well-approximated as a conformal map to nonlinear order.

Supplementary Note 3. ENERGETICS OF HINGE DEFORMATIONS

We now aim to derive a continuum elasticity theory for the RS metamaterial. However, to consider the RS elastic material at full detail requires allowance of all possible deformations of the porous elastic structure. Such a tensor field theory for deformations would be complicated and unwieldy, leaving very little room for analytic progress. Fortunately, due to the careful design of the pores, we may think of the material as composed of square elements of side length a connected by comparatively small "hinge" elements of thickness ℓ. This enables an effective description of the material as a collection of hinges, written in terms of simplified variables which prove to be very convenient for coarse-graining. We establish this effective "hinge-based" energetic formalism below.

In the limit that $\ell \ll a$, the hinge will become very flexible compared to the stiff square pieces. We therefore follow successful previous examples [2, 3] in which the large square elements are approximated as rigid bodies and all strain deformation is assumed to take place at the hinges. This assumption is readily verified in the Finite Element deformation data, where the stress is generically found to be dramatically localized to the hinge region, as shown in Supplementary Fig. 3.

To quantify the energetics in this effective description, we consider a single hinge connecting two elastic blocks as shown in Supplementary Fig. 4-a. Knowledge of the deformation tensor $F_{ij}^{\text{hinge}}(\mathbf{r})$ throughout the hinge completely determines the system configuration, as stress and strain are already assumed to be zero elsewhere. Here, $\mathbf{r}$ and $\mathbf{R}$, respectively, are target and reference coordinate systems describing the fine structure of the hinge deformations, in contrast to $\mathbf{x}$ and $\mathbf{X}$ which will describe a coarsened picture of continuum deformations of the metamaterial at the mesoscale. Given the complete constitutive relation for the elastic material, the tensor field $F_{ij}^{\text{hinge}}(\mathbf{r})$ provides sufficient information to compute the energy density at each point in space. For generality we write this simply as an unknown function $e(\mathbf{r}) = \phi(F_{ij}^{\text{hinge}}(\mathbf{r}))$.

We now confine our focus to hinges with aspect ratio ~ 1, which is the case for the experiments and simulations in this manuscript. This condition generally removes opportunities for bistability and other path-dependent elastic behavior at the hinge. Therefore, prescribing the orientations and positions of the two squares attached to a particular hinge will leave only one possible force-balanced deformation $F_{ij}^{\text{hinge}}(\mathbf{r})$ in the hinge. The energy of this hinge may therefore be rewritten as a function of the two square orientations and two 2d vector positions, six numbers altogether. Accounting for translational and rotational symmetry leaves only three scalar parameters necessary to describe the configuration of the hinge. We will focus on two distinct, yet convenient choices for these three parameters as described below.

Importantly, we note that arbitrary relative positions and orientations of a pair of squares will generally cost a great deal of energy and may stretch the hinge to the point of fracture. We wish to focus on low-energy configurations that do not stretch the hinge too much, which includes a particular set of nonlinear rearrangements due to the design based on an ideal-hinge mechanism. Therefore, in order to find an appropriate parametrization for these low-energy configurations, we must first quantify the finite-hinge (finite-energy) analogue of this mechanism. As shown in Supplementary Fig. 4a,b, fixing the distance between the square centers (c) and allowing the square orientations to relax defines our twisting angle $T(c) \equiv \pi/4 - \psi(c)/2$. Matching with the common nomenclature for twisting in kagome and RS lattices, this represents the amount the squares must each be rotated away from the fully extended state to balance torque at fixed distance. To twist the squares oppositely according to $T(c)$ while moving the square centers to distance c therefore constitutes our generalized mechanism. In the limit that the hinge size ℓ goes toward zero, and the strain is increasingly localized to an infinitesimal hinge, this motion must approach the ideal frictionless hinge version of the mechanism. We may therefore write the twisting function as

$$T(c) = \text{ArcCos}\left(\frac{c}{\sqrt{2}a}\right) + \frac{\ell}{a}\delta_T(c) + \mathcal{O}(\ell/a)^2 \tag{13}$$

where $\delta_T(c)$ is an unknown dimensionless function capturing the first-order corrections to the twisting due to the finite size and stiffness of the hinge. Eventually we will proceed to ignore this first order correction, but must acknowledge that the function $\delta_T(c)$ may diverge in the vicinity of $c = \sqrt{2}a$ (the untwisted state). Both our hinge theory and subsequent coarse-graining of the Rotating Square (RS) lattice mechanics will break down near this untwisted state and we therefore leave analysis of this anomalous point in the configuration space for future work.

Alternatively, we may explore this mechanism by fixing the orientation $\psi = \pi/2 - 2T$ and allowing the positions to relax to a square center spacing $c(T)$ which can be expressed as

$$c(T) = \sqrt{2}a\text{Cos}(T) + \ell\delta_c(T)\,. \tag{14}$$

Again, the second term on the right-hand side is a correction term dependent on an unknown function δ_c which will eventually be neglected in the small hinge limit.

We now use these mechanism twisting functions to define two parametrizations of the hinge energy. In both cases, we first choose to move to a point along the mechanism pathway, setting the distance c and twisting angle to $\psi = \pi/2 - 2T(c)$ as shown in Supplementary Fig. 4 (left). We may then deviate from this configuration in two ways: In the first, we may choose to fix the square orientations and allow a small vector deviation $\delta\mathbf{c}$ in the relative positions as shown in the top right of Supplementary Fig. 4. We will refer to this as the "vector parametrization". Alternatively, we could keep the square centers fixed in place and allow each to have a small reorientation away from the mechanistic twisting orientation as shown in Supplementary Fig. 4. This defines deviation angles $\delta\theta_A$ and $\delta\theta_B$ and we will refer to this as the "angular parametrization". In the end, the choices will span the same configurations of the hinge structure.

Both parametrizations above are useful for isolating the "non-mechanistic" part of the hinge motion into small variables. It is clear for small hinges that comparatively large $\delta\mathbf{c}$ will put the hinge under large strains which scale roughly as $\sim \frac{|\delta c|}{\ell}$ and will put conventional materials at risk of tearing and fracture. We therefore work in the limit $\frac{|\delta c|}{\ell} \ll 1$. Equivalently, $(\delta\theta_A, \delta\theta_B) \ll 1$ are the appropriate conditions in the angular parametrization. We will see below that the vector parametrization is useful for

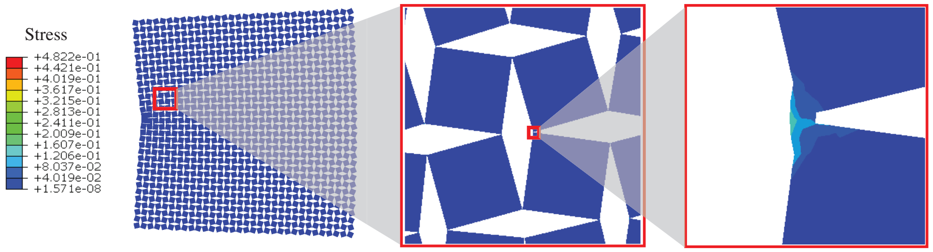

Supplementary Figure 3. Sample deformations from Finite Element Simulations displaying the detail of the elastic mesh method as well the characteristic localization of the stress (and therefore also strain) magnitude to a small region in the vicinity of the hinge.

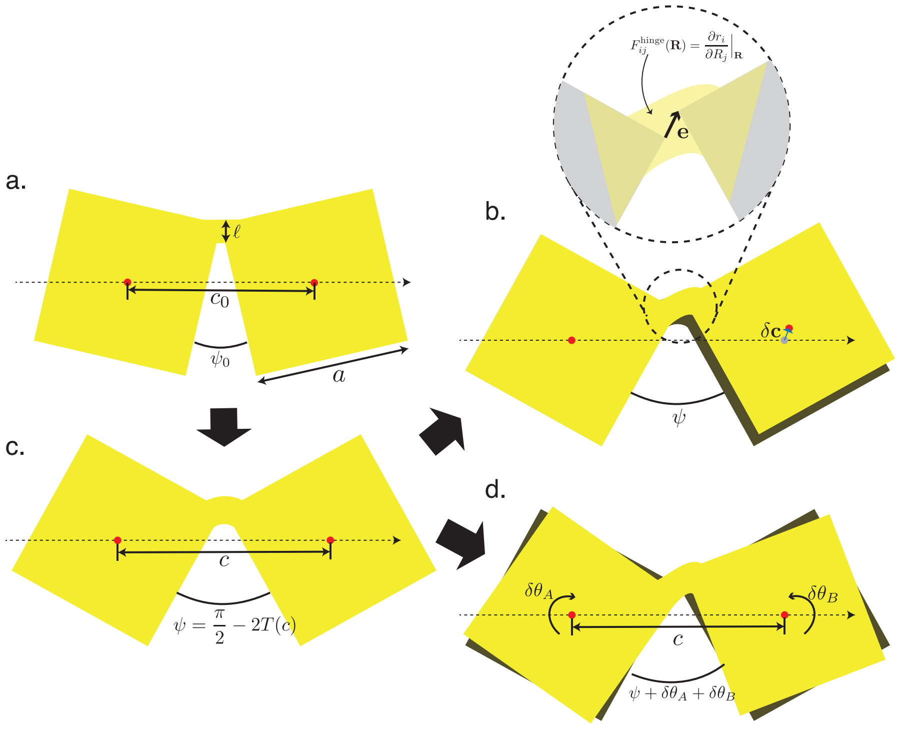

Supplementary Figure 4. In the absence of additional forces applied near the hinge, the elastic hinge energetics may be parametrized with three numbers describing the relative orientations and positions of the squares. First, the "mechanism" portion (**a** $\to$ **c**) may be traversed by fixing the center-to-center distance $c_0 \to c$ of the squares and allowing the square orientations to relax. Then small deviations around this state are allowed by either (**d**) preserving the square distance and allowing small orientation changes $(\delta\theta_A, \delta\theta_B)$ or (**b**) preserving the square orientations and allowing a small change $(\delta\mathbf{c})$ in relative square positions.

deriving scaling relations, while the angular parametrization is useful for the coarse-graining. Given some configuration of the hinge we may now write the hinge energy in terms of the vector parameters

$$E_H = g_v(T, \delta\mathbf{c}, \ell, a, T_0)\,, \tag{15}$$

or equivalently in terms of the angular parameters

$$E_H = g_a(c, \delta\theta_A, \delta\theta_B, \ell, a, T_0)\,. \tag{16}$$

where T_0 is the "pretwist" present in the reference configuration of the hinge ensemble. Finally, we note that it is useful to be able to convert between these descriptions. Neglecting second and higher order terms in the deviations $\delta\mathbf{c}$ and dimensionless hinge size ℓ/a, we may

write

$$\delta\theta_A = -\frac{(\delta c)_x}{\sqrt{2a^2-c^2}} + \frac{(\delta c)_y}{c}$$
$$\delta\theta_B = -\frac{(\delta c)_x}{\sqrt{2a^2-c^2}} - \frac{(\delta c)_y}{c}\,. \tag{17}$$

Even with perfect knowledge of the hinge geometry and nonlinear material elasticity, finding the form of the functions g_v, g_a would require solving a difficult nonlinear mechanics problem. However, a lot can be discerned *without* knowing the exact form of the energy and instead relying only on scaling arguments. In the vector parametrization, our hinge deformation tensor is a function not only of the location within the hinge $\mathbf{x}$, but also this tensor field must depend on the inputs T and $\delta\mathbf{c}$. It is instructive to consider rescaling the hinge reference and final states by a factor γ while preserving shape. This means that points in the hinge $\mathbf{r}$ are mapped to points $\tilde{\mathbf{r}} = \gamma\mathbf{r}$ and the deformation tensor is preserved via $\tilde{F}_{ij}^{\text{hinge}}(\tilde{\mathbf{r}}) = F_{ij}^{\text{hinge}}(\mathbf{r})$. Because the deformation tensor is preserved, so is the energy density. The only difference in computing the total energy of the rescaled hinge comes from integration over a rescaled domain, and we find $\tilde{E_H} = \gamma^2 E_H$. However, this rescaling also impacts the relative locations of the large square elements. Consider the internal vector $\mathbf{e}$ which points from the projected corner of square A to the projected corner of square B as shown in the zoom in Supplementary Fig. 4-b. Due to the preserved shape of the hinge, this internal vector must also scale directly with γ. This internal vector also has an analytic form

$$\mathbf{e} = \ell\delta_c(T)\hat{x} + \delta\mathbf{c}\,, \tag{18}$$

where $\delta_c(T)$ is again the correction to the mechanism from equation (14). Considering the configurations where $\delta\mathbf{c} = 0$ in equation (18), we note that because $\mathbf{e}$ scales with γ, and because ℓ also scales directly with γ, then δ_T must not scale with γ. Noting that δ_T is independent of $\delta\mathbf{d}$, it will remain scale-free when we also apply such a displacement $\delta\mathbf{c}$. Because each side of equation (18) must scale the same way with γ, we have now established the result that the displacement vector $\delta\mathbf{c}$ indeed must scale linearly with γ.

Rescaling the hinge has no impact on the square relative orientations ψ nor on the "pre-twist" T_0. This rescaling impacts only ℓ and $\delta\mathbf{c}$ and the hinge total energy E_H as described above (in the vector parametrization). We may therefore rewrite the hinge energy function

$$E_H = g_v(T, \delta\mathbf{c}, \ell, a, T_0) = \ell^2 g_1(T, \frac{\delta\mathbf{c}}{\ell}, a, T_0)\,, \tag{19}$$

where g_1 is another unknown function, whose value may be thought of as a mean energy density in the hinge.

Another way of arriving at equation (19) is the following: given we had started with a smaller hinge of the same initial shape, we must arrive at the same final force-balanced hinge shape by simply rescaling the applied displacement vector $\delta\mathbf{c}$ by the same factor as ℓ. Therefore, we must be able to write the hinge energy as in equation (19) as a function of the current twist, the pretwist, and the value of $\delta\mathbf{c}$ relative to ℓ.

We now apply our previous assumption that we will avoid extreme, high energy configurations of the hinge where material fracture becomes likely. Quantitatively, this is the condition $\frac{\delta\mathbf{c}}{\ell} \ll 1$. We may use this condition to expand the energy in equation (19) to second order, yielding

$$\begin{aligned} E_H =& \ell^2 g_T(T) \\ &+ \frac{(\delta\mathbf{c}\cdot\hat{c})^2}{2}k_s(T) + \frac{(\delta\mathbf{c}\cdot\hat{c}_\perp)^2}{2}k_\mu(T) \\ &+ \mathcal{O}\left(\frac{\delta\mathbf{c}}{\ell}\right)^3, \end{aligned} \tag{20}$$

The twist-dependent coefficients may be written in terms of g_1 via

$$\begin{aligned} g_T(T) &= g_1(T, 0, T_0) \\ k_s(T) &= \left.\frac{\partial^2 g_1}{\partial(\delta\mathbf{c}\cdot\hat{c})^2}\right|_{T,\delta\mathbf{c}=0,T_0} \\ k_\mu(T) &= \left.\frac{\partial^2 g_1}{\partial(\delta\mathbf{c}\cdot\hat{c}_\perp)^2}\right|_{T,\delta\mathbf{c}=0,T_0}. \end{aligned} \tag{21}$$

Using equations 17 & 21, we can convert this energy to the angular parametrization. This becomes

$$\begin{aligned} E_H =& \ell^2 g(\tilde{c}) \\ &+ \frac{a^2 k_1(\tilde{c})}{2}(\delta\theta_A^2 + \delta\theta_B^2) + a^2 k_2(\tilde{c})\delta\theta_A\delta\theta_B \\ &+ \mathcal{O}\left(\frac{\delta\mathbf{c}}{\ell}\right)^3, \end{aligned} \tag{22}$$

where $\tilde{c} = \frac{c}{\sqrt{2}a}$ is the dimensionless rescaling of the square center distance and the moduli are defined by

$$\begin{aligned} g(\tilde{c}) =& g_T(T(\sqrt{2}a\tilde{c})) \\ =& g_T(\text{ArcCos}(\tilde{c}\text{Cos}(T_0))) \\ k_1(\tilde{c}) =& \frac{\text{Sin}(T(\sqrt{2}a\tilde{c}))^2}{2}k_s(T(\sqrt{2}a\tilde{c})) \\ &+ \frac{\text{Cos}(T(\sqrt{2}a\tilde{c}))^2}{2}k_\mu(T(\sqrt{2}a\tilde{c})) \\ k_2(\tilde{c}) =& \frac{\text{Sin}(T(\sqrt{2}a\tilde{c}))^2}{2}k_s(T(\sqrt{2}a\tilde{c})) \\ &- \frac{\text{Cos}(T(\sqrt{2}a\tilde{c}))^2}{2}k_\mu(T(\sqrt{2}a\tilde{c}))\,. \end{aligned} \tag{23}$$

While the function g_1 remains unknown, and the energies in equations 16 & 15 may not be evaluated directly, these expressions provide useful insight into the nonlinear macroscopic energetics of the RS metamaterial while excluding extremal configurations, as shown in Supplementary Note 4. Importantly, as the function g_1 defined

in equation (19) is scale-free, there is no remaining "hidden" dependance on the hinge size ℓ nor the square size a in the energy function and the scaling is directly apparent.

We have therefore avoided a complicated tensor-field description of our structured elastic metamaterial, in favor of a hinge-based energetic formalism based on a greatly simplified set of degrees of freedom: the positions and orientations of the square elements. In this formalism, energetics and scaling permit analytic descriptions which prove helpful for coarse graining.

Supplementary Note 4. COARSE-GRAINING PROCEDURE FOR NONLINEAR NONUNIFORM MECHANICS OF THE ROTATING SQUARE LATTICE

We now derive the continuum elasticity theory for the rotating-square (RS) lattice, as presented in the main text. Following Supplementary Note 3, we approximate the elastic RS metamaterial as a collection of rigid square elements connected by comparatively flexible hinges. The hinges and square elements may be made out of the same material, while the comparative flexibility arises due to the small size of the hinge.

We establish the local continuum energy penalty created by a mapping $\mathbf{x} \to \mathbf{X}(\mathbf{x})$. This mapping will control the location of the square centers, while the orientations are left free to realize torque balanced configurations. Following the main text result that soft deformations of the RS metamaterial are near-conformal, we restrict our focus to deformations composed of a large nonlinear dilation, a finite local rotation, and a small generic shear. This prescription is captured by the deformation tensor

$$F_{ij}(\mathbf{x}) = R(\phi(\mathbf{x}))_{ik}\alpha(\mathbf{x}) \left[\delta_{kj} + \frac{s_1(\mathbf{x})}{2}\sigma^{(3)}_{kj} + \frac{s_2(\mathbf{x})}{2}\sigma^{(1)}_{kj}\right] \tag{24}$$

where

$$\sigma^{(1)} = \begin{bmatrix} 0 & 1 \\ 1 & 0 \end{bmatrix} \quad \& \quad \sigma^{(3)} = \begin{bmatrix} 1 & 0 \\ 0 & -1 \end{bmatrix} \tag{25}$$

are Pauli matrices and $\mathbf{R}(\phi)$ a standard rotation by ϕ. To understand this tensor, we break it down piece by piece. First, recall that by definition this tensor describes the Jacobian of the deformation map $\mathbf{X} \to \mathbf{x}(\mathbf{X})$ via $F_{ij} = \frac{\partial x_i}{\partial X_j}$. The infinitesimal material vectors are therefore understood to transform under deformation via $\mathrm{d}\mathbf{X} \to \mathrm{d}\mathbf{x} = \mathbf{F} \cdot \mathrm{d}\mathbf{X}$. Consider, then, the action of each element of $\mathbf{F}$ going from right to left, in the order that they act on $\mathrm{d}\mathbf{X}$. The first (rightmost, in square brackets) term applies a linear strain composed solely of shears $s_1(\mathbf{X})$ and $s_2(\mathbf{X})$ defined as

$$\begin{aligned} s_1 &= \partial_x u_x - \partial_y u_y \\ s_2 &= \partial_x u_y + \partial_y u_x \end{aligned} \tag{26}$$

where u_i is the i-th component of displacement, defined at this intermediate deformation for the purposes of understanding. Defined in this way, s_1 and s_2 correspond to the traditional notions of pure shear and simple shear, respectively. Following the application of the linear shear, the material vectors undergo an isotropic dilation $\alpha(\mathbf{X})$ and are then rotated by $\phi(\mathbf{X})$, bringing us to the final configuration. While this is a general form for such a (large dilation plus small shears) deformation, we note that these new variables ϕ, α, s_1, s_2 cannot vary arbitrarily through space, as they must still obey a closure condition to guarantee geometric (mechanical) compatibility. This is because a generic, spatially varying candidate strain field may not correspond to a valid displacement field, just as a generic candidate electrical field does not always correspond to an electrostatic potential. In the vanishing shear limit the closure condition takes the form

$$0 = \partial_i \alpha - \alpha \epsilon_{ij} \partial_j \phi \,, \tag{27}$$

where ϵ_{ij} is the 2-by-2 antisymmetric unit tensor. Reassuringly, equation (27) captures the Cauchy-Riemann condition for a deformation to be conformal. To see this, note that the derivative of a conformal function may be written in modulus-argument form as $\partial_z f = f' = \alpha \exp[i\phi]$. Then, as conformal functions must be functions of z only, we must have $\partial_{\bar{z}} f' = 0$. Writing out the real and imaginary parts of this condition leads to the x- and y-components of equation (27).

We now choose the vectors $\mathbf{c}_\nu$ connecting neighboring square centers (and where $\nu = \{1, 2, 3, 4\}$ indexes *which* neighbor) to represent the infinitesimal vector elements of the effective continuum material, and thus F_{ij} prescribes all the relative square locations after deformation. If we can now find a similar recipe for writing down the square orientations in terms of continuum quantities, then our description of the metamaterial configuration is effectively complete. We will then be able to write down the hinge energies and sum these over the unit cell to get a continuum energy density. To identify the torque-balanced square orientations, we note that there are only two squares per unit cell. We therefore assume that square orientations are controlled by two angular fields which vary slowly through space. A unit cell contains squares A and B which after deformation are located at $\mathbf{r}_A$ and $\mathbf{r}_B$ and have orientations θ_A and θ_B. There is already some intuition for the expected orientations in terms of the counter-rotation mechanism of the squares arising from the local lattice dilation factor $\alpha(\mathbf{X})$. This is expressed by writing the orientations as

$$\begin{aligned} \theta_A &= T(\sqrt{2}a\alpha(\mathbf{r}_A)) + \phi(\mathbf{r}_A) + \Delta T(\mathbf{r}_A) + \Delta\phi(\mathbf{r}_A) \\ \theta_B &= -T(\sqrt{2}a\alpha(\mathbf{r}_B)) + \phi(\mathbf{r}_B) - \Delta T(\mathbf{r}_B) + \Delta\phi(\mathbf{r}_B) \,, \end{aligned} \tag{28}$$

where $T()$ is the same mechanism function from Supplementary Note 3, while $\Delta T, \Delta\phi$ are the two smooth fields required to capture the two orientation degrees of freedom in each unit cell. Note that while equation (28) relies on a well-motivated guess, there is no loss of generality

due to the presence of the correction fields $\Delta T, \Delta\phi$, and conveniently that if our guess is correct, then enforcing torque balance will simply set the value of these fields locally to zero. In the limit of vanishing shears and vanishing gradients of α and ϕ, we find that ΔT and $\Delta\phi$ also vanish and therefore the magnitude of these angular correction fields must be on the order of the shears or $\nabla\phi$ or smaller.

We are now equipped with a sufficient description to specify the energy (equation 22) of an arbitrary hinge connecting the square initially centered at $\mathbf{X}$ to the square initially centered at $\mathbf{X}+\mathbf{c}_\nu^0$ in terms of our continuum quantities $(\phi, \alpha, s_1, s_2, \Delta T, \Delta\phi)$. Constructing the required geometric quantities to evaluate the hinge energy, we first find via application of the deformation tensor that the length of $\mathbf{c}_\nu$ changes as

$$|\mathbf{c}_\nu| \to |\mathbf{c}_\nu|\alpha(\mathbf{x}+\frac{1}{2}\mathbf{c}_\nu{}^0)\times \left[1+\frac{s_1(\mathbf{x})\left[(\mathbf{c}_\nu^0\cdot\hat{e}_1)^2-(\mathbf{c}_\nu^0\cdot\hat{e}_2)^2\right]}{2|\mathbf{c}_\nu^0|^2} + \frac{s_2(\mathbf{x})(\mathbf{c}_\nu^0\cdot\hat{e}_1)(\mathbf{c}_\nu^0\cdot\hat{e}_2)}{|\mathbf{c}_\nu^0|^2}\right], \tag{29}$$

where $\hat{e}_1, \hat{e}_2$ are the orthogonal basis vectors (x- and y-directions, respectively) in the reference space. The deformation also produces a reorientation of this local infinitesimal via

$$\mathrm{Arg}(\mathbf{c}_\nu) \to \mathrm{Arg}(\mathbf{c}_\nu) + \phi\left(\mathbf{x}+\frac{1}{2}\mathbf{c}_\nu\right) + \frac{s_2(\mathbf{x})\left[(\mathbf{c}_\nu\cdot\hat{e}_1)^2-(\mathbf{c}_\nu\cdot\hat{e}_2)^2\right]}{2|\mathbf{c}_\nu|^2} - \frac{s_1(\mathbf{x})(\mathbf{c}_\nu\cdot\hat{e}_1)(\mathbf{c}_\nu\cdot\hat{e}_2)}{|\mathbf{c}_\nu|^2}. \tag{30}$$

This information, combined with equation (28), allows us to write the angles of deviation

$$\begin{aligned}\delta\theta_A^{(\nu)} =& \Delta T + \Delta\phi - \frac{\partial T}{\partial\alpha}\mathrm{Re}\left[c_\nu\partial_z\alpha\right] - \mathrm{Re}\left[c_\nu\partial_z\phi\right] \\ &- \frac{\mathrm{Re}[c_\nu^2]}{2|c_\nu|^2}\left[\frac{\partial T}{\partial\alpha}\alpha s_1 + s_2\right] + \frac{\mathrm{Im}[c_\nu^2]}{2|c_\nu|^2}\left[s_1 - s_2\frac{\partial T}{\partial\alpha}\alpha\right] \\ \delta\theta_B^{(\nu)} =& \Delta T - \Delta\phi + \frac{\partial T}{\partial\alpha}\mathrm{Re}\left[c_\nu\partial_z\alpha\right] - \mathrm{Re}\left[c_\nu\partial_z\phi\right] \\ &+ \frac{\mathrm{Re}[c_\nu^2]}{2|c_\nu|^2}\left[s_2 - \frac{\partial T}{\partial\alpha}\alpha s_1\right] - \frac{\mathrm{Im}[c_\nu^2]}{2|c_\nu|^2}\left[s_1 + s_2\frac{\partial T}{\partial\alpha}\alpha\right]\end{aligned} \tag{31}$$

which will be input to determine the energy of hinge ν. For convenience, we have moved to the complex formulation rather than the vector formulation where $z \equiv X_1 + iX_2$, and similar for vector quantities such as c_ν. Our continuum energy density may now be constructed by summing the hinge energy equation (16) over the four hinges in the unit cell via

$$\Phi(z) = \frac{1}{A_{\mathrm{cell}}}\sum_{\nu=1}^{4} E_H^{(\nu)} \tag{32}$$

where $A_{\mathrm{cell}} = 4a^2\cos(T_0)$ is the area of the unit cell in the reference space and $E_H^{(\nu)}$ is the energy from equation (22) for hinge ν. Inserting equations 31 and performing the sum yields

$$\begin{aligned}\Phi(z) =& \frac{\ell^2}{a^2}\frac{g(\alpha)}{\cos(T_0)^2} + \frac{(k_1(\alpha)+k_2(\alpha))T'(\alpha)^2\alpha^2}{4\cos(T_0)^2}s_1^2 + \frac{(k_1(\alpha)-k_2(\alpha))}{4\cos(T_0)^2}s_2^2 \\ &+ \left[\frac{a^2k_1(\alpha)}{4}(\frac{1}{\alpha^2}+T'(\alpha)^2) + \frac{a^2k_2(\alpha)}{4}(\frac{1}{\alpha^2}-T'(\alpha)^2)\right]|\nabla\alpha|^2 + \frac{1}{\cos(T_0)}\left(k_1(\alpha)+k_2(\alpha)\right)\left(\Delta T^2+\Delta\phi^2\right).\end{aligned} \tag{33}$$

The energy in equation (33) includes all terms which are not higher order in either ℓ/a or the shears or $|\nabla\alpha|$. To reduce to an elasticity theory in terms of spatial deformation variables, we minimize equation (33) with respect to the internal degrees of freedom ΔT and $\Delta\phi$. This leads to $\Delta T = 0$ and $\Delta\phi = 0$, which indicates that our mechanism-based guess for the square orientations in equation (28) was correct. The continuum elastic theory for the RS lattice is then

$$E = \int \mathrm{d}^2\mathbf{X}\frac{1}{2}\left\{\frac{\ell^2}{a^2}M(\alpha) + a^2\tilde{M}(\alpha)|\nabla\alpha|^2 + G_1(\alpha)s_1^2 + G_2(\alpha)s_2^2\right\}. \tag{34}$$

Where the moduli

$$M(\alpha) = \frac{2g(\alpha)}{\cos(T_0)^2}, \tag{35}$$

$$G_1(\alpha) = \frac{(k_1(\alpha) + k_2(\alpha))T'(\alpha)^2\alpha^2}{2\cos(T_0)^2}, \tag{36}$$

$$G_2(\alpha) = \frac{(k_1(\alpha) - k_2(\alpha))}{2\cos(T_0)^2}, \tag{37}$$

$$\tilde{M}(\alpha) = \frac{k_1(\alpha)}{2}\left(\frac{1}{\alpha^2} + T'(\alpha)^2\right) + \frac{k_2(\alpha)}{2}\left(\frac{1}{\alpha^2} - T'(\alpha)^2\right), \tag{38}$$

capture the energy cost of dilation, pure shear, simple shear, and dilation gradients, respectively. Note that in the small hinge limit, the strain gradient modulus may be inferred from the shear moduli via the simple relation

$$\tilde{M}(\alpha) = \frac{1 - \alpha^2\cos^2(T_0)}{\alpha^4}G_1(\alpha) + \frac{\cos^4(T_0)}{1 - \alpha^2\cos^2(T_0)}G_2(\alpha), \tag{39}$$

where we have used the analogue for the mechanism in equation (13) to replace

$$T'(\alpha) = \frac{-\cos(T_0)}{\sqrt{1 - \alpha^2\cos^2(T_0)}} \tag{40}$$

excluding higher order terms proportional to ℓ/a.

Supplementary Note 5. SYMMETRY-BASED CONSTRUCTION OF THE ENERGY FUNCTIONAL

Here, we present an alternate derivation of an energy functional of the form equation (34) without recourse to the exact RS microstructure, based instead upon its fundamental symmetries.

We consider, then, a general elastic system that:

- has a dilational mechanism
- has standard elastic translational and rotational symmetries
- has a four-fold rotational symmetry
- has an $x \to -x$ mirror symmetry
- has a $y \to -y$ mirror symmetry (as implied by the previous two symmetries)

These last three properties arise from the *p4g* wallpaper symmetry group, which applies to all states of the lattice along the uniform dilational mechanism.

	s_1	s_2	$\partial_x\alpha$	$\partial_y\alpha$
C_4	$-s_1$	$-s_2$	$-\partial_y\alpha$	$\partial_x\alpha$
x-mirror	s_1	$-s_2$	$-\partial_x\alpha$	$\partial_y\alpha$
y-mirror	s_1	$-s_2$	$\partial_x\alpha$	$-\partial_y\alpha$

TABLE I. Action of symmetry operations on deformation fields

A uniform application of the mechanism will generate an energy density that we again label $(\ell/a)^2M(\alpha)$, having identified the scaling with hinge thickness in Supplementary Note 3. Additionally, energy terms may arise from other components of the deformation tensor: pure shear (s_1), simple shear (s_2) and rotation (ϕ). However, rotation in particular can be easily eliminated: the system is invariant under a uniform rotation and equation (27) (Cauchy-Riemann) shows that for dilation-dominated deformations, rotation gradients can be expressed in terms of dilation gradients instead (up to a small correction from the shears). Overall, our energy density can therefore be expressed in terms of three fields: (s_1, s_2, α) and gradients thereof. In general, this allows for terms proportional to $s_1^2, s_1s_2, \partial_x\alpha\partial_y\alpha$ etc. Terms such as $\partial_x s_1\partial_y s_1$ and $s_2\partial_x^2\alpha$ are also permitted, but these will be higher order in gradients and shears which are small due to the comparative size of the unit cell and design based on a mechanism, respectively. Note that, for present convenience, we are using notation different from the previous section, where $x \to X_1$ and $y \to X_2$.

Such energy terms are only permitted if they are invariant under the discrete symmetries of the undeformed or uniformly dilated lattice: a four-fold rotation about the center of a square and two mirror symmetries about the center of an open pore.

The action of the symmetry operations on the relevant fields are listed in Table. I. Enforcing that the energy remain the same before and after applying the x-mirror symmetry eliminates (s_2, $\partial_x\alpha$, s_1s_2, $\partial_x\alpha\partial_y\alpha$, $s_2\partial_y\alpha$, $s_1\partial_x\alpha$) from the energy density. Similarly, the y-mirror symmetry eliminates the terms ($\partial_y\alpha$, $s_2\partial_x\alpha$, $s_1\partial_y\alpha$). Finally, the C_4 symmetry eliminates S_1, and further enforces that the energetic coefficient of the $(\partial_x\alpha)^2$ term match the coefficient of $(\partial_y\alpha)^2$. We do not consider terms involving second gradients of the dilation field, which are ruled out in the coarse-grained microscopic theory.

With this, the energy functional to lowest order in the small shears and dilation gradients must take the form

$$E = \int d^2\mathbf{x}\frac{1}{2}\left\{\frac{\ell^2}{a^2}M(\alpha) + a^2\tilde{M}(\alpha)|\nabla\alpha|^2 + G_1(\alpha)s_1^2 + G_2(\alpha)s_2^2\right\}. \tag{41}$$

where the coefficients have been named to connect to the coarse grained theory in equation (34).

Importantly, while we have not invoked the specific nature of the RS mechanism or material properties to

derive this elastic energy, is it specific to the point symmetry group of the lattice and a different energetic form is expected for other dilational metamaterials such as the kagome lattice.

Supplementary Note 6. STRESS OF NEAR-CONFORMAL DEFORMATIONS OF THE RS METAMATERIAL

Having constructed the Energy in equation 34, we would like to know the corresponding stress tensor, as this is the more common quantity used in nonlinear elasticity. However, this theory is both nonlinear and includes strain gradient terms, and we require a relation which appropriately incorporates these effects. To do this, we start with the essential information that the functional derivative with respect to displacement gives the force density as

$$f_i = -\frac{\delta E}{\delta u_i}\,. \tag{42}$$

In addition, we know that the force density is the divergence of a stress tensor

$$f_i = \partial_j N_{ji}\,, \tag{43}$$

and so the process of deriving the stress is a matter of taking the functional derivative in equation 42 and fitting it into the form of equation 43. Our energy functional is an integral over an energy density which is a function of our quantities α and s_1 and s_2. These are strain quantities, and may be expressed in terms of the Lagrangian strain

$$\varepsilon_{ij} = \frac{1}{2}(\partial_i u_j + \partial_j u_i + \partial_i u_k \partial_j u_k) = \frac{1}{2}(C_{ij} - \delta_{ij})\,, \tag{44}$$

where $C_{ij} = F_{ki}F_{kj}$ is the right Cauchy-Green deformation tensor (i.e. the metric of deformation) and $F_{ij} = \partial_j u_i$ is the deformation gradient tensor. The variables in our energy functional equation 34 may be written, to lowest order in s_1, s_2, in terms of this strain via

$$\alpha = \sqrt{\det[F]} = (\det[C])^{1/4} = (\det[2\varepsilon + \mathbb{1}])^{1/4} \tag{45}$$

$$s_1^2 = \frac{1}{2}\frac{\mathrm{Tr}\left[C\cdot\sigma^{(3)}\right]}{\sqrt{\det\left[C\right]}} = \frac{\mathrm{Tr}\left[\varepsilon\cdot\sigma^{(3)}\right]}{\sqrt{\det\left[2\varepsilon - \mathbb{1}\right]}} \tag{46}$$

$$s_2^2 = \frac{1}{2}\frac{\mathrm{Tr}\left[C\cdot\sigma^{(1)}\right]}{\sqrt{\det\left[C\right]}} = \frac{\mathrm{Tr}\left[\varepsilon\cdot\sigma^{(1)}\right]}{\sqrt{\det\left[2\varepsilon + \mathbb{1}\right]}}\,. \tag{47}$$

Therefore, we should think of our energy functional as having the form

$$E = \int \mathrm{d}^d x \Phi(\varepsilon_{ij}, \partial_k \varepsilon_{ij}) \tag{48}$$

With this, taking the functional derivative in equation 42 is a matter of using the chain rule for functional derivatives

$$\frac{\delta E}{\delta u_l(x)} = \int \mathrm{d}^d x' \frac{\delta E}{\delta \varepsilon_{ij}(x')}\frac{\delta \varepsilon_{ij}(x')}{\delta u_l(x)}\,. \tag{49}$$

Here, ϵ_{jk} may be thought of as a functional of u_i and this derivative is taken using the definition of the functional derivative

$$\frac{\delta F[u]}{\delta u(x)} = \lim_{h\to 0}\frac{F[u(x') + h\delta(x-x')] - F[u(x')]}{h} \tag{50}$$

which becomes

$$\begin{aligned}\frac{\delta \varepsilon_{ij}[u(x')]}{\delta u_l(x)} &= \lim_{h\to 0}\frac{\varepsilon_{ij}[u(x') + h\hat{e}_l\delta(x-x')] - \varepsilon_{ij}[u(x')]}{h} \\ &= \frac{\partial \epsilon_{ij}(x)}{\partial(\partial_k u_l(x))}\partial_k \delta(x-x')\,.\end{aligned} \tag{51}$$

And using the form of equation 44, we find

$$\frac{\delta \epsilon_{ij}[u(x')]}{\delta u_l(x)} = \frac{1}{2}\left(\delta_{lj}\partial_i + \delta_{il}\partial_j + \partial_i u_l \partial_j + \partial_j u_l \partial_i\right)\delta(x-x')\,. \tag{52}$$

Plugging this back into equation 49, and simplifying a bit, we may find

$$N_{ij} = \frac{\delta E}{\delta \epsilon_{ik}} F_{kj}\,. \tag{53}$$

where

$$\frac{\delta E}{\delta \epsilon_{ij}} = \frac{\partial \Phi}{\partial \epsilon_{ij}} - \partial_k \frac{\partial \Phi}{\partial(\partial_k \epsilon_{ij})} \tag{54}$$

is the standard functional derivative. As the divergence of this stress in terms of the reference space coordinates gives the force density in *reference* space, this construction captures the Nominal stress (also known as Engineering stress and corresponding to the transpose of the first Piola-Kirchhoff stress) in accordance with the nonlinear elasticity literature [4]. Using the standard conversion formulae taking us between the different standard nonlinear stress definitions, we find that the second Piola-Kirchhoff stress is given by the simple expression

$$S_{ij} = \frac{\delta E}{\delta \epsilon_{ij}}\,. \tag{55}$$

Inserting the actual energy from equation 34, we will find

$$\begin{aligned} S_{ij} &= \left[T_1\right]\delta_{ij} \\ &\quad - s_1\left[T_1 - \frac{G_1}{\alpha^2} - \frac{a^2}{\alpha s_1}\tilde{M}\partial_k\alpha\partial_k s_1\right]\sigma^{(3)}_{ij} \\ &\quad - s_2\left[T_1 - \frac{G_2}{\alpha^2} - \frac{a^2}{\alpha s_2}\tilde{M}\partial_k\alpha\partial_k s_2\right]\sigma^{(1)}_{ij}\,, \end{aligned} \tag{56}$$

where

$$\begin{aligned} T_1 = &-\frac{a^2}{\alpha}\left[\tilde{M}\partial_k\partial_k\alpha + \tilde{M}'\partial_k\alpha\partial_k\alpha\right] - 2\frac{a^2}{\alpha^2}\tilde{M}\partial_k\alpha\partial_k\alpha \\ &+ \frac{1}{2\alpha}\Bigg[\frac{\ell^2}{a^2}M' + a^2\tilde{M}\partial_k\alpha\partial_k\alpha \\ &\qquad + \frac{s_1^2}{2}\left(G_1' - 4\frac{G_1}{\alpha}\right) + \frac{s_2^2}{2}\left(G_2' - 4\frac{G_2}{\alpha}\right)\Bigg]\,. \end{aligned} \tag{57}$$

While the 2nd Piola-Kirchhoff doesn't admit a direct physical interpretation in terms of real traction forces across material surfaces, it is mathematically convenient and can easily be converted to the more physically relevant Cauchy stress with the formula

$$\sigma_{ij} = \frac{1}{\det[F]} F_{ik} S_{kl} F_{jl} \,. \tag{58}$$

Supplementary Note 7. ANALYTIC PREDICTION OF DEFORMATION

Having identified the effective continuum energy which will govern the deformation of the RS metamaterial, we would like to use it to predict material response to applied loads. Taking the limits of small hinge and small lattice spacing in equation (34) leads to an energy which is comparatively very stiff against deformations that include shears, and the shear terms dominate the energy functional. In this limit, the material will choose to expel shear whenever possible, leading directly to the conformal maps (which definitionally exclude local shear) as the lowest energy deformations. With only shear terms in the energy, the conformal deformations form a highly degenerate space of ground states. The point displacements applied in the Finite Element simulations from Supplementary Note 1 are not sufficient to fully break the degeneracy — an infinite space of deformations satisfy the (point) boundary conditions without any shears, leaving no single prediction for the deformation.

To break this degeneracy, we turn to perturbation theory, adding back in the dilation $M(\alpha)$ and dilation gradient $\tilde{M}(\alpha)$ terms. Predicting the deformation then becomes a search for the conformal map which minimizes the perturbative energy

$$E \sim \Delta E \equiv \int \mathrm{d}\mathbf{x} \frac{1}{2} \left\{ \frac{\ell}{a} M(\alpha) + a^2 \tilde{M}(\alpha) |\nabla\alpha|^2 \right\} \,. \tag{59}$$

Shown in Supplementary Fig. 5-a, this reduced form is a reasonable approximation to the true energy, with the shear energy roughly an order of magnitude smaller at small hinge size. This energy gap will be even more significant, and the perturbative approximation better, for larger material samples with more unit cells which are closer to the continuum limit. As described in the main text, this process of obtaining and using this effective theory constitutes our **conformal elasticity**.

7.1. Analytic prediction of linear deformation using effective conformal theory

The energy in equation (59) is highly nonlinear and minimization requires prior knowledge of the nonlinear stiffnesses $k_1(\alpha), k_2(\alpha), g(\alpha)$, which are not easily obtained. Even with knowledge of these moduli, analytic solutions to such nonlinear mechanics problems are hard to come by. However, taking the theory to lowest order in strains and rewriting in terms of a complex displacement $u(z) = f(z) - z = u_x(z) + iu_y(z)$ yields

$$E = \int \mathrm{d}z\mathrm{d}\bar{z} \left\{ K \mathrm{Re}(\partial_z u)^2 + \frac{1}{2}\tilde{K}|\partial_z^2 u|^2 \right\} + E_{constr} \tag{60}$$

where $\partial_z = 0.5(\partial_x - i\partial_y)$ is a standard complex derivative [1] and

$$\begin{aligned} K &= \frac{\ell^2 g''(1)}{4a^2 \cos(T_0)^2} \\ \tilde{K} &= a^2 \tilde{M}(\alpha = 1) \end{aligned} \tag{61}$$

are the bulk and bulk gradient moduli, respectively. Note the unknown function g, defined in Supplementary Note 3, which captures the mechanism energy. In order to incorporate the applied point motions (constraints), Lagrange multiplier terms have been added to the energy functional as

$$E_{constr} = \int \mathrm{d}z\mathrm{d}\bar{z} \left\{ \sum_k \lambda_k (u_k - u(z_k)) \right\} \,, \tag{62}$$

where $\{u_k\}$ are the displacements prescribed at locations $\{z_k\}$.

The energy in equation (60) will be used to select between conformal displacements u which may be written as an expansion $u(z) = \sum_n C_n z^n$. As higher coefficients C_n generate sharper and sharper features, we can cut off the expansion and then solving the equations of energy minimization is reduced to a linear algebra problem.

The above is not yet sufficient to generate predictions, as the moduli K and $\tilde{K}$ remain undetermined. Conveniently, the coarse-graining procedure from Supplementary Note 4 offers a relation between these moduli which is exact in the limit of small hinges

$$\tilde{K} = a^2 \left(\sin(T_0)^2 G_1(\alpha = 1) + \frac{\cos(T_0)^2}{\tan(T_0)^2} G_2(\alpha = 1) \right) \,. \tag{63}$$

With this, the strain level moduli (K, G_1, G_2) obtained numerically in the methods section of the main text constitute all necessary information to make linear deformation predictions. For simulations with hinge thickness $\ell_0 = 0.1\mathrm{mm}$ these moduli are $(K, \tilde{K}) = (35\mathrm{Pa^*m}, 125\mathrm{Pa^*m^3})$. To find predictions at different hinge thicknesses ℓ, we rely on the coarse graining results, which indicate that $K \sim \ell^2$ while all other moduli $G_1, G_2, \tilde{K}$ are independent of ℓ. Therefore, we write $K(\ell) = \frac{\ell^2}{\ell_0^2} K(\ell_0)$ in terms of the known $K(\ell_0)$ and use these values to solve the linear algebra problem for the coefficients $\{C_n\}$ and therefore our prediction for $u(z)$. These predictions capture all but a small portion Δ^2_{pred} of the error, as displayed in Supplementary Fig. 5-b. This completes

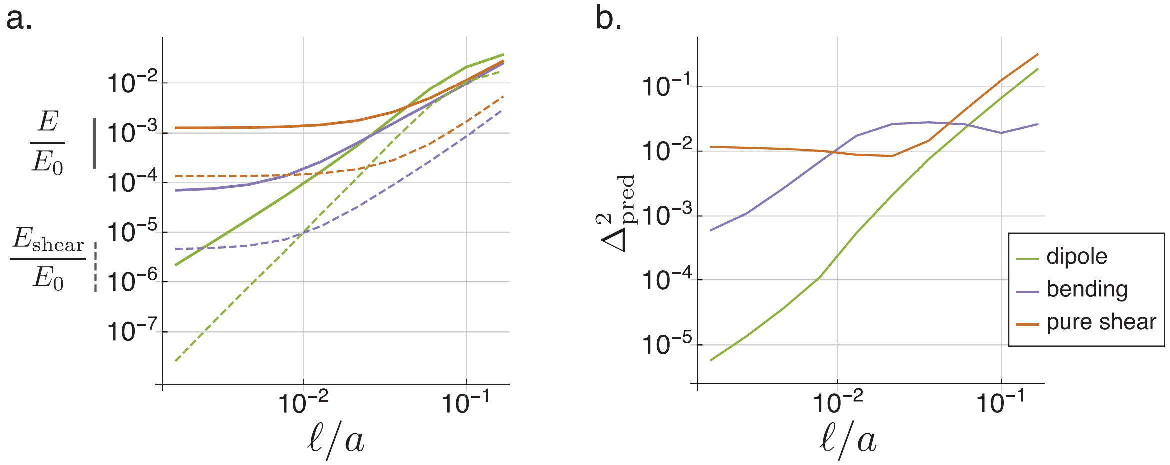

Supplementary Figure 5. (**a**) We show that going from equation (34) to equation (59) is a good approximation by showing that the contribution to the energy from the shear terms is an order of magnitude smaller (or more) in the limit of small hinges. Energies are normalized by E_0, which is the amount of energy required to create the same coarse strain magnitude in the absence of the pore structure, and which showcases also the comparative softness of the porous metamaterial. The energy in equation (59) may also be used to generate predictions of small deformations which have very small fractional mean squared error shown in (b).

Supplementary Note 8. LAGRANGE MULTIPLIER APPROACH TO CONFORMAL ELASTICITY

In this Section, we employ a more conventional method of enforcing the shear-free conditions on the RS metamaterial by the introduction of Lagrange multipliers λ_1, λ_2. This produces insight into the shear stresses enforcing this shear-free condition. For simplicity, we consider a system without gradient terms, so that the energy takes the form

$$E = \int d^2\mathbf{R}\ [b(J) + \lambda_1(F_{11} - F_{22}) + \lambda_2(F_{12} + F_{21})]\,, \tag{64}$$

where F_{ij} is the deformation tensor, $J = \alpha^2$ is the determinant of its matrix form and the general dependence on the reference coordinate has been suppressed.

The conditions for equilibrium are that the energy cannot be lowered by any movement of the material from its target position, $\mathbf{r}(\mathbf{R}$. This can be expressed as a functional derivative (See, e.g., Lazar and Kirchner [5] or Saremi and Rocklin [6]):

$$\frac{\delta E}{\delta r_1(\mathbf{R}')} = \frac{\delta E}{\delta r_2(\mathbf{R}')} = 0. \tag{65}$$

These functional derivatives inherit many properties of conventional derivatives and in particular if the energy depends on the position only through an intermediate function, such as the deformation tensor, we have the chain rule:

$$\frac{\delta E}{\delta r_i(\mathbf{R}')} = \int d^2\mathbf{R}''\, \frac{\delta E}{\delta F_{jk}(\mathbf{R}'')} \frac{\delta F_{jk}(\mathbf{R}'')}{\mathbf{r}(\mathbf{R}')}. \tag{66}$$

Consequently, after some algebra, we obtain our equilibrium conditions:

$$b''(J)\,[F_{11}\partial_1 J + F_{12}\partial_2 J] + \partial_1\lambda_1 + \partial_2\lambda_2 = 0, \tag{67}$$
$$b''(J)\,[F_{11}\partial_2 J - F_{12}\partial_1 J] - \partial_2\lambda_1 + \partial_1\lambda_2 = 0. \tag{68}$$

In obtaining this expression, we have already used that $F_{22} = F_{11}, F_{21} = -F_{12}$ (the constraints). To this, we now add our compatibility condition, as discussed in the Methods section of the main text. We find that this compatibility condition is equivalent to

$$(\partial_1 + i\partial_2)(F_{11} - iF_{12}) = 0, \tag{69}$$

or

$$\partial_{\bar{z}} f' = 0 \tag{70}$$

where

$$z \equiv x + iy, \tag{71}$$
$$\partial_{\bar{z}} = \frac{1}{2}(\partial_x + i\partial_y) \tag{72}$$
$$f' \equiv F_{11} - iF_{12}. \tag{73}$$

We now note that $J = |f'|^2$ and we can take our two original real equilibrium conditions and add the first

equation to the second equation multiplied by i, and obtain the equivalent complex condition

$$b''(J)|f'^2|\bar{f}'' + \partial_z \lambda = 0, \tag{74}$$

$$\lambda(z,\bar{z}) \equiv \lambda_1 + i\lambda_2. \tag{75}$$

governing the constrained equilibrium states. Thus, there are stresses that are supported even in the absence of deformation ($f'(z) = 0$): the structure supports shear stresses that are functions of $\bar{z}$ only. That is, they are antiholomorphic, whereas the low-energy deformations are holomorphic, or conformal. In addition, there is an additional shear stress proportional to $b''(J)$ that forms when there is a nonzero bulk modulus and a spatially varying deformation tensor. Similar results arise more generally when dilation gradient terms are included, still allowing for an undetermined antiholomorphic space of shear stresses.

Supplementary Note 9. BOUNDARY METHOD FOR ANALYTIC PREDICTION OF CONFORMAL DEFORMATION

As described in the main text, materials in the conformal limit admit a novel boundary method for analytic control of deformation. Here we give more explicit analytic recipes both for continuous analytic description of the metamaterial and for a discrete approximation.

As noted in the main text, a planar conformal deformation may be described by a complex analytic function $z \to f(z)$. The function $f(z)$ obeys $\partial_{\bar{z}} f = 0$ ($\bar{z}$ is the complex conjugate of z), and as a result admits well defined z-derivatives everywhere in its domain and can be expanded in a series as in equation (8). These analytic properties are preserved when the derivative is taken $f' \equiv \partial_z f$ and when we take the logarithm, defining $g \equiv \ln(f')$. Note that the logarithm has the potential to introduce nonanalyticity, yet avoiding nonphysical material configurations such as inversion and collapse into a point avoids any such problems. The function f' may be written in modulus-argument form as

$$f'(z) = \alpha(z,\bar{z})\exp(i\phi(z,\bar{z}))\,. \tag{76}$$

In finite strain theory, f' captures the deformation tensor, the function α will capture the local isotropic rescaling of area $\mathrm{d}A \to \alpha^2 \mathrm{d}A$ due to deformation, and ϕ captures the coarse material rotation (e.g. reorientation of the entire unit cell). Taking the logarithm, our function g may be written

$$g = \ln(\alpha) + i\phi\,. \tag{77}$$

The real part of this function now depends only on the scaling factor α while the imaginary part depends only on the rotation ϕ. It is well known that the real and imaginary parts of a complex analytic function each separately satisfy the Laplace equation, which appears as $4\partial_z\partial_{\bar{z}}\ln(\alpha) = 0$ in complex form for the real part of g. The Laplace equation admits a unique solution given Dirichlet data (the local value of $\ln(\alpha)$) all along a closed boundary. Solving this for the real part of g, the boundary data for $\ln(\alpha)$ comes from local lattice dilations along the boundary. Therefore, given prescribed dilation data all along the boundary of a closed material domain, the dilations throughout the bulk are determined by the Laplace equation.

Next we note that full knowledge of $\mathrm{Re}[g]$ across the domain may be used to infer the full analytic function g up to a purely imaginary constant. Recipes to infer g such as the method of Milne-Thompson [7] or that applied by John d'Angelo in Ref. [8] rely on the Cauchy-Riemann equations. Employing the method of John d'Angelo we may write the full function

$$g(z) = 2\ln\left(\alpha\left(x \to \frac{z}{2}\,,\, y \to \frac{z}{2i}\right)\right) + i\phi_0 \tag{78}$$

in terms of the internal dilation field $\alpha(x,y)$ up to an undetermined material rotation ϕ_0. Finally, taking the exponential $f' = \exp(g)$ yields a complete prediction of the deformation tensor throughout the bulk. Integrating f' along a path from z_i yields a prediction for the relative final position of the material point initially located at z_f

$$f(z_f) - f(z_i) = \int_{z_i}^{z_f} \exp\left[g\right] \mathrm{d}z\,. \tag{79}$$

Importantly, the conformal property guarantees that this integral is not path dependent.

In summary, the full recipe for inferring the spatial mapping $f(z)$ from the boundary is as follows:

- solve for $\alpha(x,y)$ using the boundary conditions and the equation $\nabla^2 \ln(\alpha) = 0$
- insert to determine the function $f'(z) = \exp\left[2\ln(\alpha(\frac{z}{2}, \frac{z}{2i})) + \phi_0 i\right]$ where ϕ_0 is the undetermined global rotation.
- integrate to find $f(z)$

We remark that the analytic viability of this recipe depends on the ability to generate solutions to the Laplace equation on the chosen domain, and to integrate them. The examples of square and circular domains provide convenient solvable examples. However, the true strength of our method lies in the reverse application. Given a desired conformal deformation $f(z)$, this may be prescribed by simply taking the derivative and evaluating the magnitude at the boundary, yielding the appropriate boundary dilation pattern to actuate. It is the uniqueness of the inverse recipe shown here (up to overall translations and rotations) which guarantees only the target map can satisfy these boundary conditions.

Finally, the particular choice of mechanism sets limitations to the amount the metamaterial can be dilated and contracted to avoid self intersection and material overextension. For instance, the RS mechanism requires α

to be everywhere confined to the range $1/\sqrt{2} < \alpha < 1$, when starting from the fully dilated configuration. However, very conveniently, it turns out that any sufficiently smooth choice of boundary dilations which do not over- or under-dilate the RS lattice on the boundary, will also generate a conformal deformation in the interior which does not go beyond the valid dilation range of the specific mechanism. This is due to the maximum modulus principle which states that both the maximum and minimum dilations of a conformal map will occur at the boundary.

9.1. Discrete Inference Method

For most material domain shapes and boundary dilation patterns, a closed-form solution for $f(z)$ is not guaranteed. We therefore illustrate an alternative scheme that allows for quick and accurate inference of a map from discrete boundary data, and which generates the predictions in main text Fig. 4.

In this method, we first identify the boundary via a set of discrete points $\{z_k\}$ and the corresponding local dilations $\{\alpha_k\}$. Points should be densely spaced compared to the lengthscale of variation of α_k. And, naturally, they should approximately trace a single continuous path enclosing a simply-connected region of the plane. Following this, we choose a cutoff N in the number of coefficients of

$$g(z) = \sum_{n=0}^{N-1} C_n z^n. \tag{80}$$

N should be significantly less than half the number of boundary points M in order to avoid overfitting.

Next we demand that our function $g(z)$ satisfy the boundary conditions by minimizing the error

$$\text{err} = \sum_k^M (\text{Re}[g(z_k)] - \ln(\alpha_k))^2 . \tag{81}$$

Inserting equation (80), we minimize this error with respect to the coefficients $C_n = A_n + iB_n$ yielding the equations

$$\begin{aligned} 0 =& \frac{\partial[\text{err}]}{\partial A_l} = f_l^{(A)} + \sum_n^N A_n Q_{ln} + \sum_n^N B_n R_{ln} \\ 0 =& \frac{\partial[\text{err}]}{\partial B_l} = f_l^{(B)} + \sum_n^N A_n S_{ln} + \sum_n^N B_n T_{ln} , \end{aligned} \tag{82}$$

where

$$\begin{aligned} Q_{ln} =& \sum_k^M \left(r_k^{n+l}\cos(n\theta_k)\cos(l\theta_k)\right) \\ R_{ln} =& -\sum_k^M \left(r_k^{n+l}\sin(n\theta_k)\cos(l\theta_k)\right) \\ S_{ln} =& -\sum_k^M \left(r_k^{n+l}\cos(n\theta_k)\sin(l\theta_k)\right) \\ T_{ln} =& \sum_k^M \left(r_k^{n+l}\sin(n\theta_k)\sin(l\theta_k)\right) \\ f_l^{(A)} =& -\sum_k^M \left(\ln(\alpha_k) r_k^l \cos(l\theta_k)\right) \\ f_l^{(B)} =& \sum_k^M \left(\ln(\alpha_k) r_k^l \sin(l\theta_k)\right) , \end{aligned} \tag{83}$$

and we have expressed $z_k = r_k e^{i\theta_k}$ in a complex polar form. equation (82) reduces the inference of g to a linear algebra problem which may be readily solved using built-in tools in, e.g., Mathematica. Note importantly that the row and column corresponding to $B_0 = \phi_0$ (the undetermined global rotation) are zero throughout. This row-column pair is, in general, the only one that needs to be removed before numerically solving.

The above yields the coefficients C_n of $g(z)$, which is inserted into the exponential function to yield a prediction for $\partial_z f$ and therefore constitutes a full prediction of the deformation tensor everywhere inside the domain. In order to generate the displacement predictions shown in main text Fig. 3, we employ the built-in numerical integrators in Mathematica.

Supplementary Note 10. BOUNDARY CONTROL SIMULATION PROTOCOL

To probe the viability of boundary control of soft conformal modes, we perform numerical simulations of a simplified version of the RS lattice. In this case the lattice is composed purely of Hookean springs of identical stiffness k connected at frictionless nodes. As shown in Supplementary Fig. 6, each rigid square element (grey) is emulated by a grouping of six springs. While a single cross-spring is sufficient to render a square element rigid, the inclusion of both ensures that the spring ensemble will obey the same symmetry properties as the elastic RS metamaterial.

To actuate a particular soft conformal mode, stiff springs are added at the boundary, as shown in Supplementary Fig. 6. These boundary springs are taken to have stiffness $10^4 k$, so that they will act as rigid constraints, realizing their rest lengths quite accurately compared to the soft mechanics of the bulk material. While the rest lengths in the interior (blue bonds) are chosen

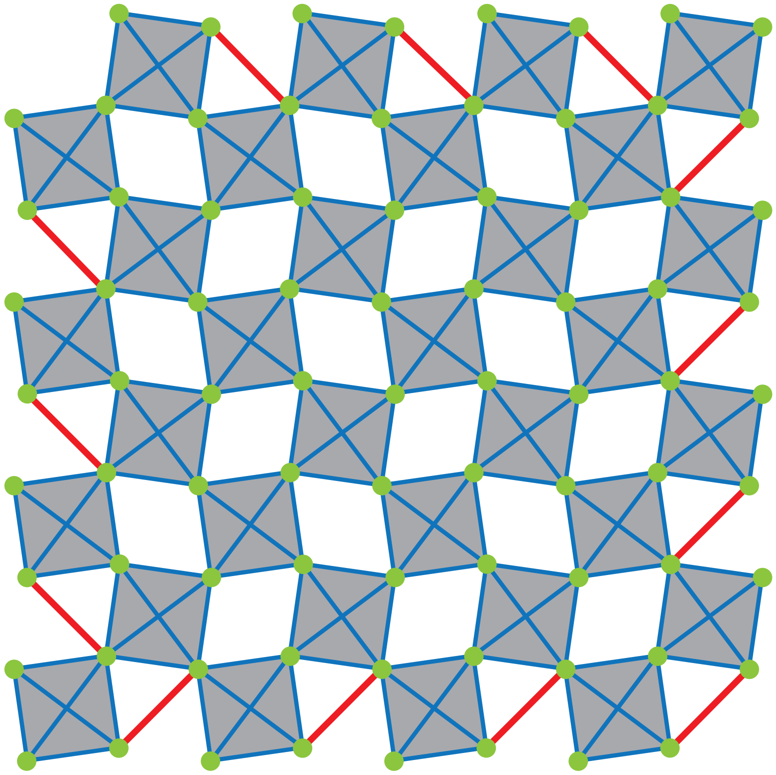

Supplementary Figure 6. A simple spring model of RS metamaterial and boundary control. The numerical method employed to investigate the viability of boundary control of the RS elastic structure is accomplished using a structure of linear springs (blue lines) connect at frictionless nodes (green dots), approximating a collection of rigid squares (grey regions, displayed for visual convenience). Additional springs are added at the boundary (red lines) and the rest lengths varied to reliably actuate soft modes as shown in the main text Fig.4.

to leave the square shape at zero energy, the rest lengths of the boundary springs are varied to match some target local dilation. For three patterns of boundary dilation, we set these rest lengths and identify force balanced states using the conjugate gradient numerical minimization procedure "minimize" from the scipy.optimize toolkit in python. At the same time, using the methods from Supplementary Note 9, and as illustrated in the main text Fig.3d-e, predictions for displacements may be generated from this input set of boundary dilations. As shown in main text Fig.4, these are in good qualitative agreement with the numerically identified force balanced configurations.

SUPPLEMENTARY REFERENCES

[1] England, A. *Complex Variable Methods in Elasticity* (Dover Publications, 2003).

[2] Coulais, C., Kettenis, C. & van Hecke, M. A characteristic length scale causes anomalous size effects and boundary programmability in mechanical metamaterials. *Nat. Phys.* **14**, 40–44 (2018).

[3] Deng, B., Tournat, V., Wang, P. & Bertoldi, K. Anomalous Collisions of Elastic Vector Solitons in Mechanical Metamaterials. *Phys. Rev. Lett.* **122**, 044101 (2019).

[4] Ogden, R. W. *Non-linear Elastic Deformations.* Dover Civil and Mechanical Engineering (Dover Publications, 1997).

[5] Lazar, M. & Kirchner, H. O. The Eshelby stress tensor, angular momentum tensor and scaling flux in micropolar elasticity. *Int. J. Solids Struct.* **44**, 4613–4620 (2007).

[6] Saremi, A. & Rocklin, Z. Topological Elasticity of Flexible Structures. *Phys. Rev. X* **10**, 011052 (2020). 1908.07499.

[7] Milne-Thomson, L. M. On the Relation of an Analytic

Function of z to Its Real and Imaginary Parts. *Math. Gaz.* **21**, 228 (1937).
[8] Stone, M. & Goldbart, P. *Mathematics for Physics: A Guided Tour for Graduate Students* (Cambridge University Press, 2009).
