# Supplementary material for: Conformal elasticity of mechanism-based metamaterials
Source: Nat Commun. 2022 Jan 11;13:211. doi: 10.1038/s41467-021-27825-0 (PMC8752823; doi:10.1038/s41467-021-27825-0)
Supplement: Supplementary file 3 — Description of Additional Supplementary Files [file 41467_2021_27825_MOESM3_ESM.pdf]

Description of Additional Supplementary Files

File Name: Supplementary Movie 1
Description: Timelapse of quasistatic foot loading experiment.

File Name: Supplementary Movie2
Description: Timelapse of quasistatic bridge loading experiment.

File Name: Supplementary Movie 3
Description: An animation depicting force-balance simulations in which actuation springs (red) are applied at the boundary of the RS metamaterial (blue squares), and activated incrementally to achieve a series of target nonlinear conformal deformations (black points).
